# Analysis of the leakage of gene repression by an artificial TetR-regulated promoter in cyanobacteria

**DOI:** 10.1186/s13104-015-1425-0

**Published:** 2015-09-19

**Authors:** Hsin-Ho Huang, Christian Seeger, U. Helena Danielson, Peter Lindblad

**Affiliations:** Department of Chemistry - Ångström, Science for Life Laboratory, Microbial Chemistry, Uppsala University, P.O. Box 523, 751 20 Uppsala, Sweden; Department of Chemistry - BMC, Uppsala University, P.O. Box 576, 751 23 Uppsala, Sweden

**Keywords:** Cyanobacteria, *Synechocystis*, TetR binding, Promoter, SPR, DNA breathing dynamics, EPBD model

## Abstract

**Background:**

There is a need for strong and tightly regulated promoters to construct more reliable and predictable genetic modules for synthetic biology and metabolic engineering. For this reason we have previously constructed a TetR regulated L promoter library for the cyanobacterium *Synechocystis* PCC 6803. In addition to the L03 promoter showing wide dynamic range of transcriptional regulation, we observed the L09 promoter as unique in high leaky gene expression under repressed conditions. In the present study, we attempted to identify the cause of L09 promoter leakage. TetR binding to the promoter was studied by theoretical simulations of DNA breathing dynamics and by surface plasmon resonance (SPR) biosensor technology to analyze the kinetics of the DNA–protein interactions.

**Results:**

DNA breathing dynamics of a promoter was computed with the extended nonlinear Peyrard–Bishop–Dauxois mesoscopic model to yield a DNA opening probability profile at a single nucleotide resolution. The L09 promoter was compared to the L10, L11, and L12 promoters that were point-mutated and different in repressed promoter strength. The difference between DNA opening probability profiles is trivial on the TetR binding site. Furthermore, the kinetic rate constants of TetR binding, as measured by SPR biosensor technology, to the respective promoters are practically identical. This suggests that a trivial difference in probability as low as 1 × 10^−4^ cannot lead to detectable variations in the DNA–protein interactions. Higher probability at the downstream region of transcription start site of the L09 promoter compared to the L10, L11, and L12 promoters was observed. Having practically the same kinetics of binding to TetR, the leakage problem of the L09 promoter might be due to enhanced RNA Polymerase (RNAP)-promoter interactions in the downstream region.

**Conclusions:**

Both theoretical and experimental analyses of the L09 promoter’s leakage problem exclude a mechanism of reduced TetR binding but instead suggest enhanced RNAP binding. These results assist in creating more tightly regulated promoters for realizing synthetic biology and metabolic engineering in biotechnological applications.

## Background

Transcription regulation plays a major role in controlling gene expression and consequently in altering biochemical reactions catalyzed by these gene products. Hence, it has high impact in synthetic biology and metabolic engineering [1–3]. The regulation relies on DNA–protein interactions between a transcription factor and a promoter [4, 5]. Strong and tightly regulated promoters are the prerequisite to realize modular control in synthetic biology [6], and genetic control in metabolic engineering [7].

Previously, we developed a TetR-regulated L promoter library for the cyanobacterium *Synechocystis* PCC 6803 and identified the L03 promoter that exhibits a wide dynamic range of regulation [8]. However, we also identified the L09 promoter as unique in showing a high leaky gene expression under repressed conditions, which is in the absence of the inducer aTc of TetR. In the present study, we attempted to understand what causes the leakage problem of the L09 promoter. Three other promoters, L10 and L11 and L12, served as controls since the four promoters differ only in a single point mutation and show different regulatory behaviors (Table 1).

**Table 1:** Promoter data from previous in vivo measurements [8]

Promoter	Point mutation^a^	Strength^b^		Induction^c^
		Induced	Repressed	
L09	C	15.6 ± 0.1	2.88 ± 0.01	5.15 ± 0.01
L10	T	17.6 ± 0.1	0.235 ± 0.003	71 ± 1
L11	G	19.1 ± 0.1	0.236 ± 0.003	77 ± 1
L12	A	0.043 ± 0.003	0.022 ± 0.003	1.9 ± 0.3

^a^The location of the point mutation in a L promoter is shown in Fig. 1

^b^The promoter strength in the induced and repressed conditions is measured by flow cytometer to detect EYFP emission in a single cell from liquid culture of cyanobacterium *Synechocystis* PCC 6803 treated with and without 100 ng mL^−1^ aTc, respectively. The unit-less promoter strength is normalized to the reference *rnpB* promoter’s strength in the respective condition

^c^The induction is the ratio of induced to repressed promoter strength (±SD)

Inspired by the observed long-range effect of a flanking single-nucleotide polymorphism on changing the binding affinity of the eukaryotic YY1 transcription factor [9], we investigated whether a flanking point mutation would have a similar effect on the binding characteristics of the L09 promoter in comparison with the L10, L11, and L12 promoters. We re-simulated the DNA breathing dynamics of the four promoters by the Extended nonlinear Peyrard–Bishop–Dauxois (EPBD) mesoscopic model to reach a single nucleotide resolution that our previous study could not achieve [8]. We also determined the kinetics of the interaction between TetR and each promoter using a surface plasmon resonance (SPR) biosensor assay.

DNA breathing refers to the transient opening of base pairs of double-stranded DNA molecules subjected to thermal motions fluctuating at physiological temperature. DNA breathing dynamics computes these spontaneous opening and re-closing events of a double stranded DNA and presents the results as a DNA opening probability profile [10]. The profile shows characteristic patterns in different probability, length, amplitude, and lifetime of DNA local transient separation (i.e. DNA bubble) [11]. A strong correlation between DNA breathing dynamics and a transcription factor binding was evidenced to predict the location of the TSS and the potential regulatory sites [12, 13]. Maintaining the integrity of the base-specific interactions in a regulatory site, base substitutions or modifications in the flanking bases might lead to suppressed or enhanced binding of a transcription factor [9, 13, 14].

Kinetic studies using SPR biosensor technology provide detailed kinetic and mechanistic insights into biomolecular interactions. DNA–protein interactions using SPR biosensor assays have been successfully applied [15], including studies of TetR–tetO interactions [16]. The assay principle is to coat a sensor surface with streptavidin and then capture biotinylated DNA via the strong biotin–streptavidin interaction [17, 18]. Once the target DNA is captured at low surface densities (20–50 RU), real-time interaction studies with the ligands of choice can be performed.

Identifying what causes leaky gene expression under repressed conditions of transcriptional regulation is important for constructing tightly regulated promoters, and consequently, for developing and realizing synthetic biology and metabolic engineering for biotechnological applications. Reasons based on simulations and experimental data to explain the leakage problem of the L09 promoter are discussed and future directions are proposed.

## Methods

### DNA fragments

**Fig. 1:**
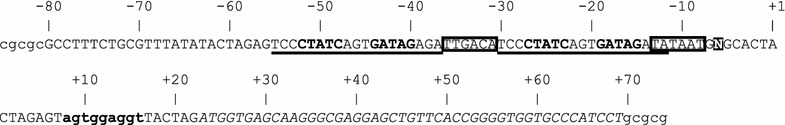
The 160 bp DNA sequence used in DNA breathing dynamics simulations and in the SPR experiments. The promoter region is from −55 to +1: the highlighted N at −6 is C, T, G, and A in the L09, L10, L11, and L12 promoter, respectively. The −35 (TTGACA) and −10 (TATAAT) elements are *boxed*. Two TetR binding sites are *underlined* and the specific bases-of-contacts in each site for TetR binding are *bold capitals*. The TSS locates at +1. The partial 3′-end of terminator BBa_B0015 is shown in the region from −80 to −64. The RBS is in *bold lowercase*. The 5′-end of the reporter *eyfp* gene is in *italic*, starting at +23. Two 8-bp and one 6-bp BioBrick scars locate from −63 to −56, from −1 to +7, and from +17 to +22. The clamp sequences in both 5′- and 3′-end are in *lowercase*

DNA fragments comprising a L promoter were prepared in length of 160 bp (Fig. 1) and biotinylated at the 5′-end of the template strand by PCR using the Phusion Hot Start II High-Fidelity DNA Polymerase (Thermo Scientific, Waltham, USA) with the forward and the biotinylated reverse primers. The forward primer is 5′-CGCGCGCCTTTCTGCGTTTATATACTAGAGTCCCTATCAGTGATAGAGATTGAC-3′.

The reverse primer is 5′-CGCGCAGGATGGGCACCACCCCGGTGAACAGCTCC-3′ and biotinylated on its 5′-end. The DNA fragments were purified by the Zymoclean Gel DNA Recovery Kit (Zymo Research, Irvine, USA) from a 1.2 % agarose gel after gel electrophoresis.

### DNA breathing dynamic simulation

Monte Carlo simulations on the Extended nonlinear Peyrard–Bishop–Dauxois (EPBD) mesoscopic model and its parameters describing DNA breathing dynamics [13] were performed with MATLAB (MathWorks, Natick, USA) using the same set [8] of 2000 different seeds of random number generator and with parallel computing with its distributed computing toolbox. A 160 bp DNA sequence (Fig. 1) containing clamp sequences on each end of a strand was simulated at 303 K with periodic boundary conditions to prevent the end effect. Each realization takes 2.1 × 10^7^ steps and as the 1 × 10^6^ steps reaches the initial equilibrium and then records every 500 steps to have 40,000 snap shots of the displacements of a base pair. Every accepted configuration in an advanced step is determined by the standard Metropolis algorithm. From 40,000 recorded displacements of each base pair in the 160 bp DNA sequence, if the displacements at a base pair and its following consecutive 3–10 base pairs are larger than 2.8 Å, it counts one opening event at the first base in the defined DNA bubble length from 4 to 11 bp. The opening probability of a base pair is the ratio of summed opening events to 40,000 recorded displacements. The DNA opening probability profile is averaged from 2000 realizations.

### SPR biosensor based interaction analysis

The SPR based interaction studies were performed on a Biacore 2000 instrument (GE Healthcare, Uppsala, Sweden) using CM5 sensor chips (GE Healthcare). Streptavidin (Sigma Aldrich, St. Louis, USA) was immobilized using amine coupling chemistry at a temperature of 25 °C and a flow rate of 5 μL min^−1^. The surface was activated by injecting 200 mM EDC and 50 mM NHS for 7 min. Streptavidin (100 μg mL^−1^ in 10 mM Na-acetate, pH 5.5) was injected for 10 min and the surface was deactivated by injecting 1 M ethanolamine, pH 8.5, for 7 min. Finally, the surface was washed with five injections of 50 mM NaOH (30 s, 30 μL min^−1^), essentially as described previously [15, 17]. The running buffer consisted of 10 mM Hepes, 150 mM NaCl, 0.05 % Tween-20, pH 7.4 (HBS-P).

Promoters (L09, L10, L11, and L12) were diluted into HBS-P to a final concentration of 10 nM and injected over streptavidin containing sensor surfaces using a flow rate of 5 μL min^−1^ until surface densities of approximately 40 RU were reached. The surface was then washed at a flow rate of 30 μL min^−1^ by injecting 1 M NaCl for 100 s followed by 0.05 % SDS for 30 s. A separate flow cell with immobilized streptavidin was used as a reference surface.

TetR (Imgen BioSciences, Fall River, USA) storage buffer was exchanged to HBS-P using Protein desalting columns (Thermo Scientific) according to the manufacturer’s instructions. For kinetic analysis, the temperature was increased to 30 °C. TetR was injected in twofold concentration series (62–4 nM) at a flow rate of 60 μL min^−1^ for 90 s. The dissociation time was 240 s. HBS-P was used as running buffer. After every TetR injection, the surface was regenerated by injecting a mixture of 1 M NaCl and 1 M MgCl_2_ for 30 s (60 μL min^−1^). For interaction studies of induced TetR, the protein was incubated with 2 uM aTc and 2 mM MgCl_2_. The dilution series was performed into HBS-P supplemented with 2 uM aTc and 2 mM MgCl_2_.

**Scheme 1:**
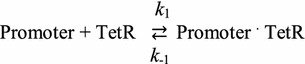
Mechanism for the reversible one-step interaction between TetR and the immobilized promoter. The interaction is described by the association rate constant (*k*_1_) and the dissociation rate constant (*k*_−1_)

Data analysis was performed using the BIAevaluation software 3.0.2 (GE Healthcare). The raw interaction data was double referenced by first subtracting the data from the reference surface, followed by subtracting the response of a blank sample. The interaction mechanism was determined by performing global nonlinear regression analysis using a reversible 1-step model (Scheme 1). The model describes the reversible interaction of TetR to the respective promoter characterized by the association rate constant (*k*_1_) and the dissociation rate constant (*k*_−1_).

The theoretical signal under saturating conditions [*R*_*max*_ (RU)] was calculated according to Eq. 1 including the molecular weights of TetR and the respective promoter [*M*_*TetR*_, *M*_*promoter*_ (g mol^−1^)], the number of binding sites on the promoter (*valency*) and the surface density of the immobilized promoter [*R*_*immob*_ (RU)].

1 $$R_{\hbox{max} } = \frac{{M_{TetR} }}{{M_{promoter} }}valency \times R_{immob}$$

## Results

### Trivial difference in DNA opening probability profiles between the L09 promoter and other L promoters

**Fig. 2:**
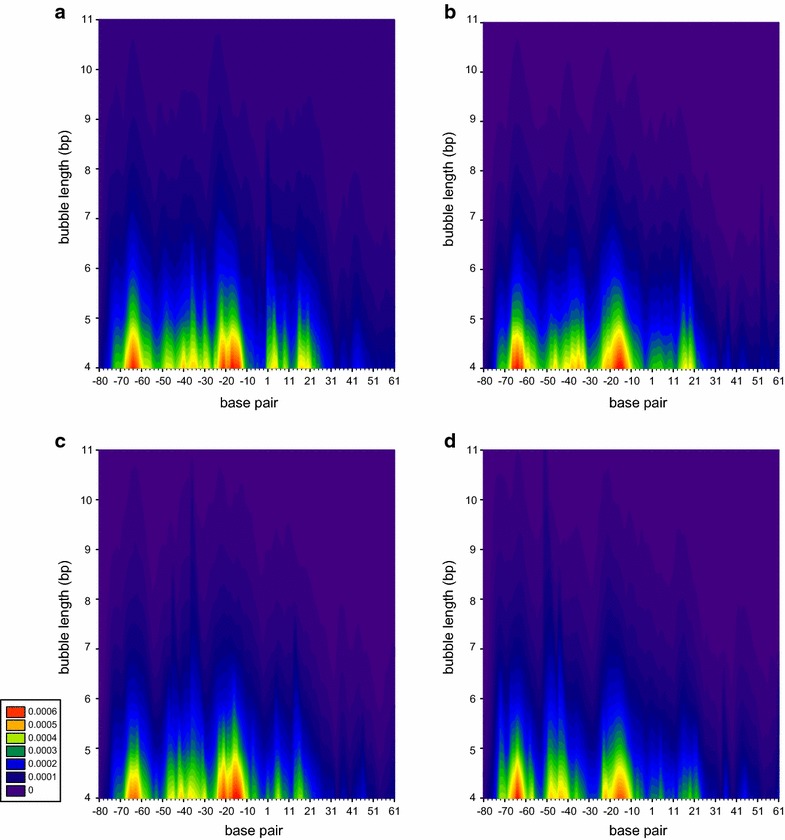
The DNA opening probability profile of L09 (**a**), L10 (**b**), L11 (**c**), and L12 (**d**) promoters simulated by the EPBD model at 303 K

To better understand the effect of a point mutation on the DNA opening probability profile, the 160 bp DNA fragments of the L09, L10, L11, and L12 promoters shown in Fig. 1 were re-analyzed with the EPBD model. The likelihood of a base pair to stretch in a selected DNA sequence depicts the DNA opening probability profile which was averaged from the accepted configurations induced by thermal fluctuations at 303 K in the defined displacing threshold as 2.8 Å and a defined DNA bubble length from 4 to 11 bp (Fig. 2). The opening probability is close to zero within the *eyfp* gene region. The characteristic peaks corresponding to the RNA Polymerase (RNAP) binding sites at the −10 and −35 elements and to two TetR binding sites at −52 to −40, and at −27 to −15 can be clearly identified. The additional peak centering at −64, the location of the partial 3′-end of the BBa_B0015 terminator, might not contribute to TetR or RNAP binding since there is no specific cognate site for binding. We conclude that four similar DNA opening probability profiles due to a single point mutation can be computed by the EPBD model, which further resolves the results obtained by the averaged PBD model [8].

**Fig. 3:**
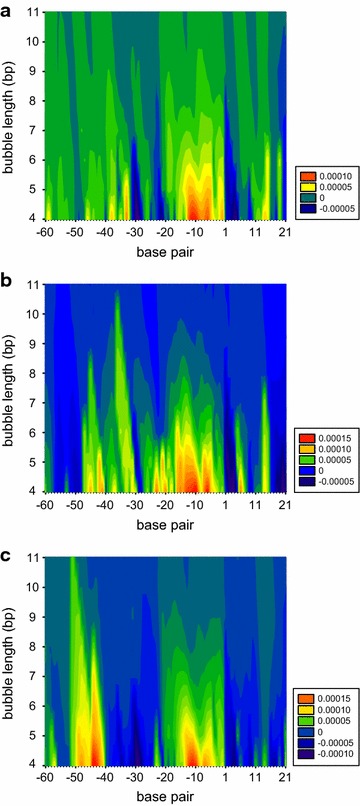
The difference between the EPBD-model-simulated DNA opening probability profiles of L09 promoter and L10 (**a**), L11 (**b**), L12 (**c**) promoters, respectively

To further distinguish these similar DNA opening probability profiles, the difference was calculated by subtracting the profile of the L09 promoter from each profile of the L10, L11, and L12 promoters (Fig. 3). The difference in probability is about ±1 × 10^−4^, corresponding to around four openings in 40,000 total counts. Although the differences are trivial, the DNA opening probabilities of the L10, L11 and L12 promoters are higher than of the L09 promoter at the positions −44 to −42 and −19 to −17, being within the two TetR binding sites. The nucleotides GAT at these positions interact with Arg28, Gln38, and Pro39 of the α3 subunit of TetR [19]. When observing the essential positions for RNAP binding and transcription initiation [20], the probabilities are higher at −12 and −6 in the L10, L11, and L12 promoters than in the L09 promoter. Adenine at −12 of the −10 element is the direct contact point for the recognition of bacterial RNAP σ subunit [21]. The nucleotide at −6 (at 2 bp immediately downstream of the −10 element) effectively affects the lifetime of the RNAP-promoter open complex [22]. However, the probabilities at the −35 element, i.e. from −36 to −31, are higher in the L10 and L11 promoters, but lower in the L12 promoter, in comparison to L09 promoter. To investigate whether these trivial differences in the DNA opening probability profile cause the unique leaky gene expression of the L09 promoter under repressed conditions, the TetR binding kinetics to the L09, L10, L11 and L12 promoters were determined using an SPR biosensor assay.

### Identical kinetic characteristics of TetR-promoter interactions

**Fig. 4:**
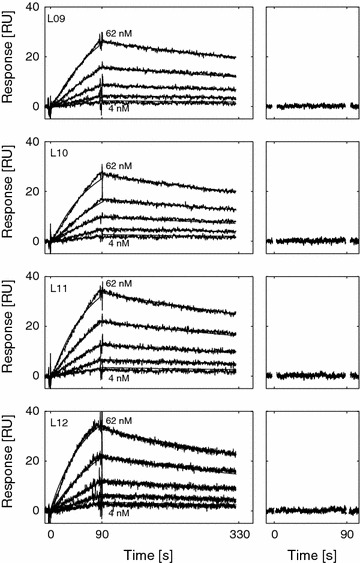
Sensorgrams of TetR interacting with promoters L09, L10, L11 and L12 in the absence (*left*) and presence (*right*) of [aTc∙Mg]^+^. TetR was injected in two-fold dilution series from 62 to 4 nM over immobilized promoters. The *solid lines* overlayed the experimental data (*left*) are theoretical best fit lines obtained by global non-linear regression analysis

In the first step of the SPR based interaction analysis, streptavidin was immobilized on SPR biosensor surfaces at surface densities of approximately 2500 RU. The promoters L09, L10, L11 and L12 were immobilized via biotin-streptavidin capture at surface densities of 30–50 RU. Clear interactions between TetR and the four promoters were detected (Fig. 4). They all displayed slow association and dissociation rates. Equilibrium was not reached within 90 s and the TetR-promoter complex did not dissociate completely within 240 s. Regeneration with 1 M NaCl and 1 M MgCl_2_ removed non-dissociated TetR quantitatively from the surface before a new cycle was started thereby maintaining practically identical surface characteristics throughout the experiment. The visual similarity of the different sensorgrams was further confirmed by global nonlinear regression analysis using a reversible 1-step interaction model (Scheme 1). The kinetic rate constants k_1_ (≈2 × 10^5^ s^−1^ M^−1^) and k_−1_ (1 × 10^−3^ s^−1^) and the equilibrium dissociation constant K_*D*_ (6 × 10^−9^ M) are identical with respect to the uncertainties of the repeated experiments for the four promoters (Table 2). The estimated R_*max*_ values (Eq. 1) indicated that almost all promoter binding sites were accessible and able to bind TetR. When TetR incubated with 2 μM aTc and 2 mM MgCl_2_ to be in its effector-bound conformation [19, 23], no interactions with the promoters were detected (Fig. 4).

**Table 2:** Kinetic parameters for the interaction between TetR and promoters L09, L10, L11, and L12

Promoter	*k*_1_ (s^−1^ M^−1^)^a^ (pk_1_)	*k*_−1_ (s^−1^)^a^ (pk_−1_)	*K*_*D*_ (M)^b^ (p*K*_*D*_)
L09	2 × 10^5^ (−5.2 ± 0.1)	1 × 10^−3^ (3.0 ± 0.1)	6 × 10^−9^ (8.2 ± 0.1)
L10	2 × 10^5^ (−5.3 ± 0.1)	1 × 10^−3^ (3.0 ± 0.1)	5 × 10^−9^ (8.3 ± 0.1)
L11	2 × 10^5^ (−5.3 ± 0.3)	1 × 10^−3^ (3.0 ± 0.1)	6 × 10^−9^ (8.2 ± 0.2)
L12	2 × 10^5^ (−5.3 ± 0.2)	2 × 10^−3^ (2.8 ± 0.1)	8 × 10^−9^ (8.1 ± 0.1)

^a^Association (*k*_1_) and dissociation rate constants (*k*_−1_) are shown together with the pk_1_ and pk_−1_ values and the respective standard deviations

^b^The equilibrium dissociation constants (*K*_*D*_) are shown together with the respective p*K*_*D*_ values and standard deviations. The data is based on three replicate experiments (±SD)

## Discussion

For developing promoters regulated in a wide dynamic range by TetR, the L promoter library was constructed previously for *Synechocystis* PCC 6803 (*Synechocysits*) [8]. A point mutation with adenine, thymine, cytosine, and guanine at 2 bp downstream of the −10 element generated the unique L09 promoter. To understand why a point mutation makes L10 and L11 promoters tightly regulated, while the L09 promoter is leaky upon TetR binding (Table 1), potential differences in DNA–protein binding were investigated. The developed SPR biosenor assay allowed to reliably measure the interactions between TetR and the promoters L09, L10, L11, and L12. The approach of capturing biotinylated DNA via covalently immobilized streptavidin has been described in detail previously [17, 18]. For this type of assay, the DNA is biotinylated at a defined position (here 5′ end of template strand) leading to a uniform orientation when immobilized on the sensor surface. The high affinity of the streptavidin–biotin interaction leads to a very stable sensor surface with practically no baseline drift. Furthermore, the structural stability of both streptavidin and the captured DNA allow interaction studies under a long period of time without a considerable loss of surface functionality. The surface density of the immobilized DNA should be reduced to a minimum to increase the accessibility of the binding sites. Different DNA capture levels have been reported, ranging from 300 to 600 RU [24] down to 0.7–3.5 RU [15]. Generally, DNA levels from 20 to 50 RU are recommended [17, 18] and these were the levels that were achieved in the present study.

The kinetic analysis of the TetR-promoter interactions shows that the association and dissociation constants and the resulting equilibrium dissociation constants are practically identical (Table 2). Kinetic studies of TetR interactions with a 40 bp tetO-containing DNA fragment have been performed previously [16]. Although a direct comparison is not valid due to the different DNA sequences, the conditions in the SPR based interaction analysis were similar and the determined affinity is in the low nanomolar range as well (≈0.2 nM).

The kinetic model that was fitted to the experimental data, a reversible 1-step model (Scheme 1), might seem inappropriate to describe the TetR-promoter interaction. There are two TetR binding sites on the promoter that would make the use of a model describing the parallel and independent interactions to those more appropriate (2-site model). However, two independent and parallel interactions that have identical kinetics lead to an overall binding curve that resembles a 1-step interaction. This is supported by comparing the theoretical *R*_max_ values with those determined by the global nonlinear regression analysis. The capture levels of the promoters were between 30 and 50 RU corresponding to theoretical *R*_max_ values in the same range assuming a valency of 2. The experimental *R*_max_ levels were in the range of 30–50 RU, thereby supporting the parallel interaction of TetR at both promoter binding sites and indicating that almost all DNA binding sites were accessible.

The dimeric structure of TetR would further suggest to use a model describing the multivalent interaction of each dimer. Previously, the mechanism of the interaction between TetR and the tetO-containing promoter was analyzed using stopped-flow measurements and appears to involve the formation of an initial complex with association rate constant of 3 × 10^8^ s^−1^ M^−1^, and the subsequent formation of a second more stable complex [25]. Whether a similar 2-step mechanism occurs during the interaction of TetR with the promoters L09, L10, L11, and L12 is not known because the DNA sequences employed are similar but not identical. Furthermore, association rate constants in the range of 1 × 10^8^ s^−1^ M^−1^ cannot be measured with the instrument employed. The fact that the reversible 1-step model (Scheme 1) gave an adequate description of the experimental data might reflect that it describes the rate limiting step of the interaction, namely the specific formation of the stable TetR-promoter complex.

Because DNA breathing dynamics simulation has shown a strong correlation with the DNA–protein interactions [12, 13], the trivial difference between DNA opening probability profiles at two TetR cognate sites (Fig. 3) might have caused different TetR binding to the L09, L10, L11, and L12 promoters. However, kinetic rate constants obtained in the SPR-based analysis are identical (Table 2). The observed trivial difference in the computed DNA opening probability does not lead to a difference in the kinetic rate constants. On the contrary, for the YY1 transcription factor, an obviously reduced opening probability at its cognate binding site is consistent to the loss of binding in the ChIP experiments due to the flanking point mutation [9]. These results together indicate that flanking point mutations in the TetR-regulated L promoters do not have long-range effects on the binding of TetR.

If TetR displays practically the same kinetic rate constants when interacting with the L10, L11, and L12 promoters, what could be the reason causing leaky gene expression by the L09 promoter? The repression in transcription regulation is due to the steric hindrance upon a repressor’s binding to its cognate site in the vicinity of the core promoter to prevent RNAP binding [4]. Therefore, under the same repression, the leakage might result from the enhanced RNAP binding to the L09 promoter. Comparing the DNA opening probability profiles in Fig. 2, L09 has a larger DNA bubble at the +2 to +4 base pairs and at the +20 base pair. These regions have a critical role in downstream interactions between RNAP and promoter [26]. Specifically, the contacts with double-stranded DNA at +2 to +4 determine the formation and stability of RNAP-downstream fork junctions complex and the length of contacts possibly exceeding to the +20 base pair also assists the formation of a promoter open complex. The binding difference in this region might cause the L09 promoter to open more easily and form a more stable RNAP-promoter open complex. This might then lead to enhanced binding of RNAP to the L09 promoter. Further SPR based studies could be performed with SigA, the major sigma factor in *Synechocystis* under normal growth conditions [27], to measure its kinetics to the L09, L10, L11, and L12 promoters and also perform competition experiments against TetR binding to the promoter. However, as discussed in [28] the RNAP is structurally different between *E. coli* and cyanobacteria as well as the sigma factors used in cyanobacteria compared to in *E. coli* making such experiments much more challenging. In addition, sigma factors in *Synechocystis* may have different selectivities to differerent nucleotide sequences in the −35 and −10 regions of specific promoter sequences [29].

Previous crystallization studies show that the conformation of the [aTc∙Mg]_2_^+^-bound TetR dimer is unable to bind DNA [19, 23]. As expected, no interaction was observed when TetR was incubated and injected together with aTc and MgCl_2_ (Fig. 4). The promoter strength under induced conditions could reveal a promoter’s ability to perform transcription (Table 1). Additionally, from the discussion in our previous study [8], the repressed promoter strength of the L12 promoter reached the detection limit and indicated that this promoter is fully repressed. Comparing the induced promoter strength 0.043 to the detection limit 0.022 (Table 1), we further confirm that the L12 promoter is an extremely weak promoter.

## Conclusions

A flanking point mutation in the L09, L10, L11, and L12 promoters neither causes significant difference in the DNA breathing dynamics at the TetR binding sites nor affects the kinetic rate constants of the interactions between TetR and the respective L promoter. The leakage problem of the L09 promoter may be due to enhanced RNAP binding. These results assist in creating more tightly regulated promoters for realizing synthetic biology and metabolic engineering in biotechnological applications.
